# Knowledge domain and emerging trends in medication literacy research from 2003 to 2024: a scientometric and bibliometric analysis using CiteSpace and VOSviewer

**DOI:** 10.3389/fpubh.2025.1598482

**Published:** 2025-06-24

**Authors:** Penghong Deng, Xiaoxia Liu, Caiyun Li, Xingping Zhu, Junli Cui, Ping Hua, Gang Chen

**Affiliations:** ^1^Nanjing Drum Tower Hospital, Affiliated Hospital of Medical School, Nanjing University, Nanjing, China; ^2^Department of Infectious Diseases, The Affiliated Dongtai Hospital of Nantong University, Dongtai, China; ^3^Department of Pharmacy, Suzhou Hospital, Xiyuan Hospital of China Academy of Chinese Medical Sciences (Suzhou TCM Hospital), Suzhou, China; ^4^Department of Pharmacy, Suzhou Traditional Chinese Medicine Hospital Affiliated to Nanjing University of Chinese Medicine, Suzhou, China; ^5^Department of Pharmacy, The Affiliated Dongtai Hospital of Nantong University, Dongtai, China

**Keywords:** medication literacy, scientometric, bibliometric, CiteSpace, VOSviewer

## Abstract

**Background:**

Medication literacy (ML) has emerged as a critical global public health concern, garnering growing scholarly attention over the past two decades. To delineate major research domains, identify evolving trends, and inform future research priorities, we conducted a scientometric analysis of the scientific literature on ML.

**Methods:**

A systematic search was performed to retrieve publications on ML from the Web of Science Core Collection, covering the period from 2003 to 2024. Scientometric analyses were executed using CiteSpace and VOSviewer to visualize and evaluate collaborative networks, including co-citation references, co-occurring keywords, and contributions by countries, institutions, authors, and journals.

**Results:**

The analysis incorporated 1,968 eligible publications. A rapidly growing trend in research interest in ML was observed, with an average annual growth rate of 46.1% in publications between 2003 and 2022. Three major research trends were identified: relationship between ML and medication adherence, the development of ML-specific assessment tools, and investigation of psychosocial factors associated with ML. The United States of America, Northwestern University, Davis Tc, and Patient Education and Counseling were identified as the most cited and influential entities within this field, representing the leading country, institution, author, and journal, respectively.

**Conclusion:**

Scientometric analysis provides invaluable insights to clinicians and researchers involved in ML research by identifying leading contributors, intellectual bases and research trends. ML is evolving from unidimensional analysis to multidisciplinary exploration of dynamic mechanisms. Future research on ML is facing significant challenges, including the exploration of adherence mechanisms, validation of digital assessment tools, and the moderating effect model of socio-psychological factors on ML.

## 1 Introduction

Global demographic aging is intensifying at an accelerated pace, with the proportion of population aged ≥60 years projected to surge from 10% in 2000 to 21% by 2050 (1). This demographic shift is fundamentally altering global disease spectrum, particularly through the escalating burden of non-communicable chronic diseases (NCDs) (2). Medication interventions, serving as the primary therapeutic approach for NCDs, critically influence disease trajectory modulation and health-related quality of life. For instance, patients diagnosed with hypertension or diabetes mellitus require long-term medication adherence to maintain stable blood pressure and glycemic control. However, suboptimal medication practices presented substantial challenges: the World Health Organization (WHO) data indicated that medication-related complications accounted for one-third of annual mortality, with associated economic losses exceeding $42 billion yearly (3). Medication non-adherence, dosing inaccuracies, and inappropriate drug utilization collectively contributed to diminished treatment efficacy, elevated hospital readmissions, therapeutic failures, healthcare system strain, and excess mortality (4–6). Numerous studies have demonstrated that these clinical and economic consequences can be prevented by strengthening medication literacy (ML) (7, 8).

Evolving from the broader concept of health literacy, ML was initially conceptualized in the 2005 UK Medication Safety Report and formally operationalized by Raynor (9) as individuals’ capacity to retrieve, interpret, and apply medication-related information for informed decision-making. Numerous studies had proposed conceptually related terms to ML, including prescription literacy (specifically addressing the comprehension of prescription information) (10), pharmacotherapy literacy (emphasizing comprehensive therapeutic decision-making capabilities) (11), and pharmaceutical literacy (focusing on the understanding of specialized pharmacological knowledge) (12). In 2018, Pouliot A. et al. proposed a widely recognized academic definition, stating that ML referred to the extent to which individuals can obtain, comprehend, communicate, calculate, and process patient-specific information regarding their medications to make informed medication and health decisions (7). Research consistently identified this competence as a key determinant of medication adherence, with higher proficiency levels correlating with improved regimen adherence (13–15). Enhanced medication-related knowledge facilitated accurate interpretation of therapeutic instructions, thereby optimizing clinical outcomes and minimizing medication risks. Conversely, deficiencies in this domain were associated with poorer cardiovascular outcomes in coronary artery disease patients and reduced functional capacity among older adult populations (4, 16). These findings collectively underscored ML as a critical mediator of patient safety and therapeutic success.

The Third Global Patient Safety Challenge “Medication without Harm” strategic plan, launched by the World Health Organization (WHO), proposed that instruments and techniques should be employed to improve patients’ medication literacy and interventions should be developed to promote patients’ knowledge of drug use (17). This initiative highlighted the fact that ML and safety was one of the main research priorities in drug safety worldwide (17). In response to the emerging health goals, ML research has captured considerable interest and attention over the past two decades. Previous studies had found a low level of ML among patients with NCDs worldwide, characterized by poor understanding of medication-related knowledge, low medication adherence, and inadequate healthcare provider engagement (18–20). In addition, numerous scholars have conducted substantial research on definitions, predictive models, current problems, assessment tools, influencing factors and interventions for ML (21–24). Despite the existing publications providing insights into specific aspects of ML, this field lacks systematic integration of cumulative knowledge and research prioritization.

The exponential expansion of scholarly output necessitates advanced analytical methodologies to map this domain’s intellectual architecture. Scientometric approaches, combining bibliometric analysis with data visualization, provide robust mechanisms for quantifying research trends and knowledge dissemination patterns—a methodological paradigm distinct from traditional systematic reviews (25, 26). Importantly, this approach establishes a systematic pathway to anticipate emerging paradigms and address complex research challenges by synthesizing interdisciplinary scientific frameworks with advanced methodological tools (27, 28). Such analyses prove particularly valuable for identifying collaborative networks, benchmarking institutional contributions, and detecting disciplinary gaps, though their application remains limited in ML research. This study’s principal aim involves conducting a longitudinal scientometric evaluation to delineate the evolution, current frontiers, and emerging directions in ML research since over the past two decades. Secondary objectives focus on characterizing international collaboration dynamics, institutional productivity patterns, and knowledge dissemination channels while identifying critical research voids requiring scholarly attention.

## 2 Methods

### 2.1 Data source

The bibliometric dataset was systematically retrieved from the Web of Science Core Collection (WOSCC), a premier research database encompassing scholarly publications across 254 subject categories (29). This database is characterized by rigorous journal selection criteria, a comprehensive citation network, and standardized bibliographic fields optimized for scientometric analysis (29, 30). Compared to other major databases, WOSCC’s extensive disciplinary coverage and high-quality data have established it as the preferred source for mainstream bibliometric analysis tools, with native compatibility in software such as CiteSpace and VOSviewer (30, 31). Additionally, this dataset’s analytical utility extends beyond conventional bibliographic metadata (authorship, institutional affiliations, geographic distributions) through its integrated citation mapping functionality and multi-layered indexing architecture, establishing it as the benchmark and classical data source for scientometric investigations (29). Within the WOSCC, the Science Citation Index Expanded (SCIE) and Social Sciences Citation Index (SSCI) were selected as primary data channels. The SCIE focuses on natural sciences, encompassing fields such as physics, medicine, and engineering, while the SSCI indexes social sciences disciplines including economics, psychology, and education. Both are important components of the WOSCC, but differ in disciplinary scope and citation patterns (29). Recognized as the gold standard for disciplinary coverage, these indices employ stringent journal inclusion criteria encompassing editorial rigor, citation impact metrics, and international diversity (29), thereby ensuring the methodological validity of our analytical framework. For this study, publications addressing ML from the SCIE and SSCI were specifically extracted.

### 2.2 Retrieval strategy and data collection

**Figure 1:**
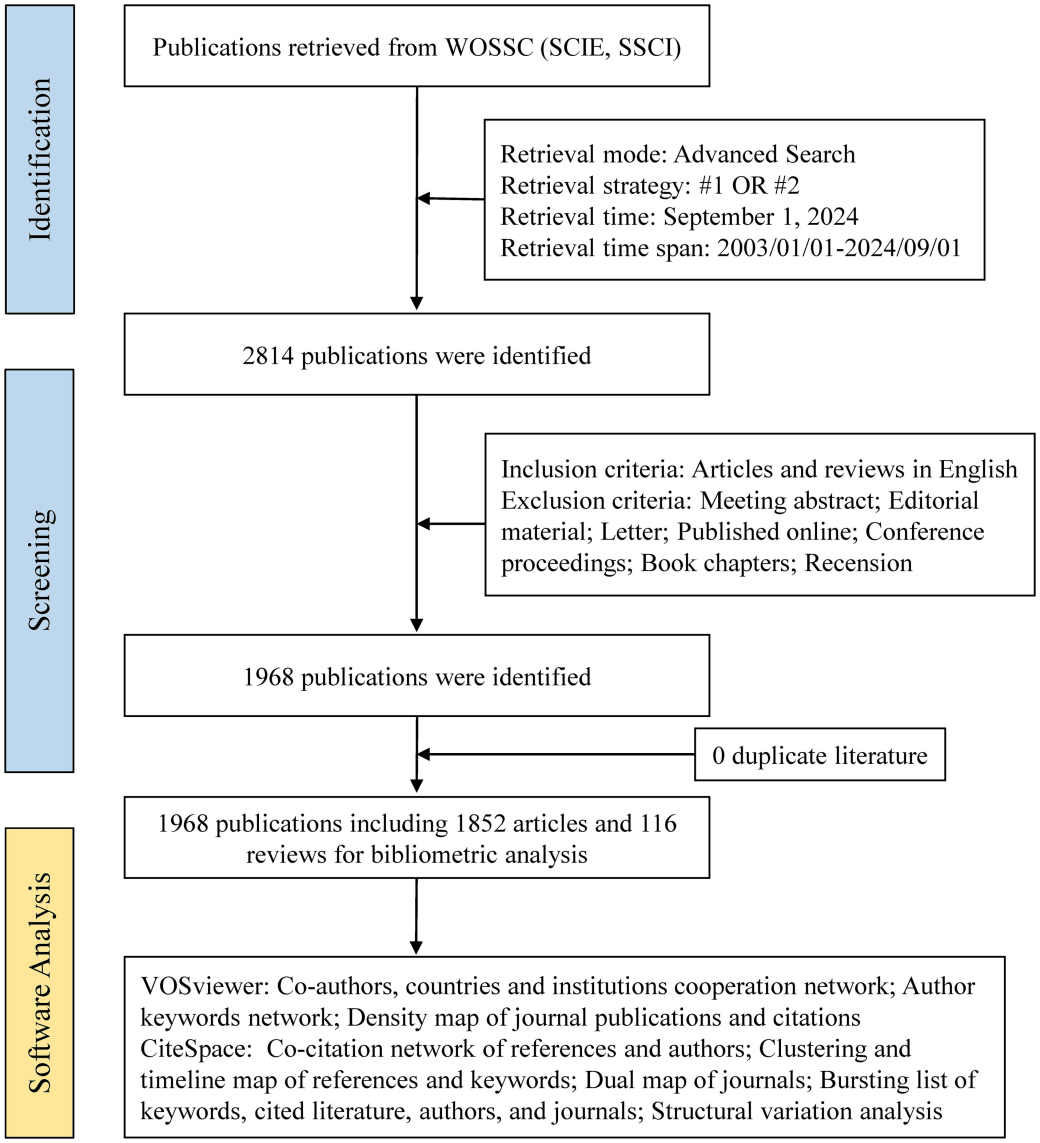
Flow chart of the scientometric study. #1: TI = (medication literacy OR drug literacy OR pharmaceutical literacy OR medication knowledge OR medication understanding OR prescription understanding OR prescription knowledge OR medication attitude OR healthy medication behavior); #2: AK = (medication literacy OR drug literacy OR pharmaceutical literacy OR medication knowledge OR medication understanding OR prescription understanding OR prescription knowledge OR medication attitude OR healthy medication behavior).

A standardized literature search was executed by a single investigator (P. D.) on September 1, 2024, to control for temporal variability in database content. The selection of core search terms was derived from both MeSH (Medical Subject Headings) terminology and the widely recognized conceptual framework in this research domain. The search strategy combined title (TI) and author keyword (AK) fields using the following Boolean parameters: (TI = (medication literacy OR drug literacy OR pharmaceutical literacy OR medication knowledge OR medication understanding OR prescription understanding OR prescription knowledge OR medication attitude OR healthy medication behavior)) OR (AK = (medication literacy OR drug literacy OR pharmaceutical literacy OR medication knowledge OR medication understanding OR prescription understanding OR prescription knowledge OR medication attitude OR healthy medication behavior)). The temporal scope encompassed January 2003 to September 2024, restricted to the original research and review papers in English. Post-retrieval processing involved implementation of predefined exclusion criteria and cross-database deduplication procedures, with the selection process visually summarized in Figure 1.

### 2.3 Measures

We employed two techniques to investigate research evolution and trends:

1. Co-citation network of references: The co-citation network is based on the relationship between two documents being cited by a third document at a specific time, representing the intellectual foundation of the third document (32). As the subject evolves, the co-citation network expands from a single network to multiple networks, illustrating the shifts in the intellectual foundation over time (32). These transitions reflect the research tracks and trends in the citing documents. By analyzing the co-citation reference network, an intellectual landscape is constructed using highly cited literature and research frontiers (identified by extracting themes from the citing literature).
2. Co-occurring network of author keywords: Keywords provide insights into the specific research areas and directly address the research hotspots within the field. The co-occurrence network measures the frequency of paired keywords within a collection of documents and captures their associations. The process of co-occurrence analysis involves extracting keywords from the documents, tallying keyword frequencies, and identifying clusters, bursts, and connections among keywords (27).

As for our secondary objectives, we constructed collaborative networks of countries, institutions, authors, and journal co-citation networks. The collaborative network incorporated countries, institutions, and authors with at least one international collaboration, enabling researchers to identify leading scholars and innovative research groups within the field. Author co-citation analysis was performed to identify highly cited authors, examine their connections, and explore the corresponding intellectual structure within the field. Additionally, journal co-citation networks help identify high-impact journals, reveal connections, and provide insights into the distribution of disciplinary knowledge domains.

### 2.4 Software and data analysis

This study employed two specialized bibliometric tools: VOSviewer (version 1.6.20) and CiteSpace (version 6.3. R3 Advanced), as documented in seminal works by van Eck & Waltman and Chen et al. (27, 33). VOSviewer, initially developed by Waltman et al. (33), provides an intuitive platform for network construction and visualization, facilitating the analysis of geographical distributions, institutional productivity, collaborative networks, and lexical co-occurrence patterns. We employed VOSviewer to analyze the networks of authors’ countries, institutions, co-author collaborations, co-occurring keywords, and density map of keywords and journals. CiteSpace, a Java-based application introduced by Chen et al. (27), specializes in emerging trends detection and knowledge domain mapping through systematic mapping, integrated bibliometric analysis, and data mining algorithms. Systematic mapping offers a comprehensive overview of existing scholarly knowledge, facilitating the identification of research domains that are sufficiently mature for meta-synthesis and those warranting further empirical investigation (27). As a quantitative analytical paradigm rooted in mathematical and statistical principles, bibliometrics enables researchers to elucidate the structural relationships and evidentiary connections within scientific literature (28). By utilizing CiteSpace, we were able to identify intellectual bases, emerging research fronts, temporal trends, and citation dynamics.

In CiteSpace, we configured the analysis with 1-year time slices. The g-index (k = 25) was employed to assess research impact, which effectively accounted for both high-cited publications and less-cited works. Clusters were groups of tightly connected nodes identified by optimizing modularity in the network, and the labeling relied on statistical likelihood to extract representative terms (27). In this study, cluster labels were derived through log-likelihood ratio (LLR) algorithmic processing of keyword corpora (*p* < 0.001). The knowledge networks generated by VOSviewer and CiteSpace comprise two fundamental elements: nodes (representing bibliographic entities including references, keywords, countries, authors, institutions and journals) and edges (denoting relational linkages through collaboration, co-citation, or co-occurrence). Node diameter correlates positively with bibliometric indicators such as citation frequency, occurrence count, or centrality metrics, serving as visual proxies for scholarly influence. Chromatic encoding of nodes and edges conveys information about the year of the corresponding citations, clusters, or occurrences. Highly connected nodes are included between and within clusters, revealing relevant areas and their evolution throughout the years.

The study employed CiteSpace’s structural variation analysis and burst detection algorithms to investigate critical factors shaping network topology and identify emerging research trajectories. Structural variation analysis quantifies the boundary-spanning potential of scholarly works through novel linkage formation metrics (34). Publications that establish interdisciplinary connections are particularly significant, as they often represent pivotal points of knowledge integration and potential catalysts for paradigm shifts (34). Complementarily, burst detection analysis, implemented through temporal data streaming algorithms, identifies citation and term patterns exhibiting sudden frequency and intensity anomalies (35). These temporal signatures frequently indicate the emergence of novel research fronts or innovations. We conducted burst detection analysis on cited references, keywords, authors, and journals to synthesize and reveal possible future research priorities. Additionally, to illustrate the evolutions and connections among clusters, we utilized timeline analysis, which spatially distributes nodes along temporal axes.

Three critical graph-theoretical indices guided cluster interpretation, following Chen et al.’s methodological framework (27): (1) Betweenness Centrality: This metric quantifies node brokerage potential through shortest-path analysis, identifying intra-cluster core nodes and inter-cluster bridging hubs. Nodes with higher centrality scores indicate their critical role within the research field, such as highly influential publications or interdisciplinary researchers. (2) Modularity (Q): This metric evaluates the tightness of intra-group connections and the separation between groups to validate the rationality of network clustering structure (Q∈[0, 1]). A Modularity value greater than 0.3 suggests that the network’s clustered structure is well-defined and meaningful. (3) Silhouette Coefficient (S): This measure assesses the homogeneity within clusters and the accuracy of node classification (e.g., whether a given publication is correctly assigned to its thematic cluster, S∈[−1, 1]), with S > 0.7 confirming substantial node similarity. Additionally, Centrality Divergence was calculated as the standard deviation of betweenness centrality distributions, serving as an indicator of structural innovation potential in boundary-spanning publications (34).

## 3 Results

Two different analytical software tools were employed to systematically map the evolution of ML research over the past two decades. This dual-method approach enabled a comprehensive evaluation of publication trends and the construction of knowledge networks, including co-cited references, author keywords, and contributions across countries, institutions, authors, and journals.

### 3.1 Analysis of publication outputs and trends

The final analysis comprised 1,968 unique scholarly publications, including 1,852 research articles and 116 review papers, which collectively accumulated 38,669 citations following screening and exclusion protocols. The authorship network encompassed 9,177 contributors, averaging 4.66 authors per publication, representing 6,732 institutions across 571 countries/territories. Temporal analysis revealed substantial growth in scholarly output, with annual publications increasing from 19 in 2003 to 194 in 2022, reflecting a compound annual growth rate of 46.1%. However, the dataset’s temporal boundary (September 2024) resulted in an apparent decline in annual publication and citation metrics. Despite relatively modest annual publications, citation impact demonstrated significant enhancement, with the average citations per document (total annual citations divided by annual publications) rising from 0.37 (7/19) in 2003 to 22.65 (4,431/178) in 2023 (Supplementary Figure S1).

### 3.2 Analysis of co-citation references

#### 3.2.1 Clusters of research

**Figure 2:**
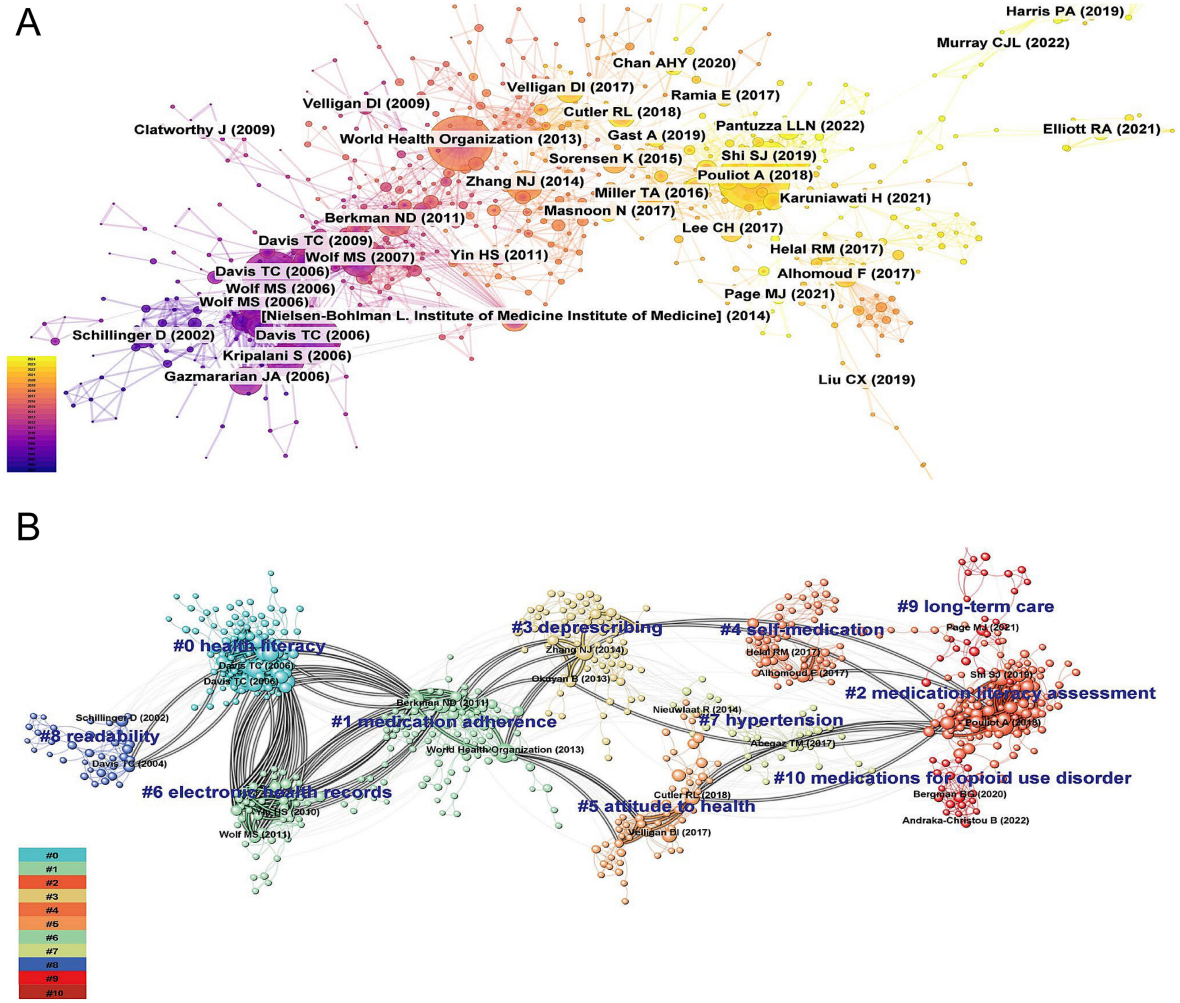
Co-citation references network **(A)** and corresponding clustering visualization **(B)** generated by CiteSpace (2003-2024). A node represents a cited reference. Each node represent one highly co-cited article. The size of a node is proportional to the co-citation count. Nodes are organized in different clusters gathered into a network of co-citation. The highlighted lines represent the evolution and connections among different clusters.

A co-citation network was conducted to identify the influential and representative research in the ML field (Figure 2A). Additionally, the co-citation reference network analysis yielded 11 distinct thematic clusters, exhibiting robust modularity (Q = 0.8259) and exceptional intra-cluster homogeneity (S = 0.9385), confirming both the credibility and distinctiveness of the groups (Figure 2B). The cluster labels were synthetically generated based on representative noun phrases extracted from the keyword lists of cited references within each cluster. More detailed descriptions of each cluster are available in Table 1. Three major research trends were identified based on the largest linkage pathways between clusters. The clusters contributing to these trends are presented with their cluster label, size, silhouette score, average year of publication, and the most representative reference.

**Table 1:** Summary of the largest clusters identified for co-citation network of references.

Cluster ID	Size	Silhouette	Mean (Year)	Top five extracted terms based on keywords
0	110	0.908	2005	health literacy; labels; safety; prescription; medication literacy
1	109	0.907	2010	medication adherence; cognition; pregnancy; medication knowledge; self-medication
2	108	0.908	2018	medication literacy assessment; relationship; structural equation model; hypertension; adherence
3	74	0.927	2014	deprescribing; polypharmacy; pharmaceutical literacy; diabetes; older adults
4	66	0.995	2017	self-medication; antibiotics; knowledge; health literacy; antibiotic
5	56	0.957	2015	attitude to health; antipsychotics; health behavior; schizophrenia; lifestyle
6	48	0.942	2009	electronic health records; ambulatory care; medication error; drug labeling; crowdsourcing
7	40	0.946	2015	hypertension; medication taking; hypertensive patients; medication history; emergency medical services
8	37	0.973	2002	readability; prescriptions; prescription drug labels; perceived effectiveness; ethnic/racial differences
9	31	0.98	2020	long-term care; medication disposal; medication adherence; pharmaceutical waste; dementia
10	18	0.996	2020	medications for opioid use disorder; acceptability; subjective norms; peer recovery specialist; treatment

The first major research trend focused on the association between ML and medication adherence. This trend emerged in 2002 with the identification of cluster #8 (*“readability”*; 37, S = 0.973, 2002) in our database, alongside a seminal article published by Schillinger et al. in JAMA, which examined the relationship between health literacy and diabetes outcomes (36). This cluster subsequently evolved into cluster #0 (*“health literacy”*; 110, S = 0.908, 2005), which served as a conceptual foundation for ML. Within this cluster, numerous studies explored the relationship between various dimensions of ML, such as literacy levels, the number of prescription medications, the understanding of prescriptions, and appropriate medication use (37, 38). Subsequently, a strong interconnection was observed between cluster 0 (*“health literacy”*) and cluster 1 (*“medication adherence”*; 109, S = 0.907, 2010) (39), suggesting a rapidly evolving phase in the exploration of the relationship between ML and adherence. Moreover, the emerging knowledge linkages between cluster #1 (*“medication adherence”*) and cluster #3 (*“deprescribing”*; 74, S = 0.927, 2014) (40) represented a new research frontier, highlighting the growing academic interest in the association between deprescribing practices and medication adherence.

The second major research trend revolved around the development of ML-specific assessment instruments. This trend began with cluster #6 (*“electronic health records”*; 48, S = 0.942, 2009) and highlighted the impact of medication administration tools and patient-centered labels on ML levels, providing a foundation for the development of subsequent assessment tools (41, 42). Over the past decade, this research field had further enriched and converged into the third largest cluster #2 (*“medication literacy assessment”*; 108, S = 0.908, 2018) (7). Within this clustering, an international definition of ML was recognized through expert consensus, significantly contributing to the standardization of ML assessment tools (7).

The third research trend focused on the investigations of psychosocial factors associated with ML. According to the inter-cluster links, clusters associated with this research topic trend were cluster #4 (*“self-medication”*; 66, S = 0.995, 2017) (43), cluster #7 (*“hypertension”*; 40, S = 0.946, 2015) (24), and cluster #10 (*“medications for opioid use disorder”*; 18, S = 0.996, 2010). Within these clusters, research participants were mainly the older adult and patients with chronic diseases, with influencing factors involving disease status, education level, economic income, and psychological cognition.

**Figure 3:**
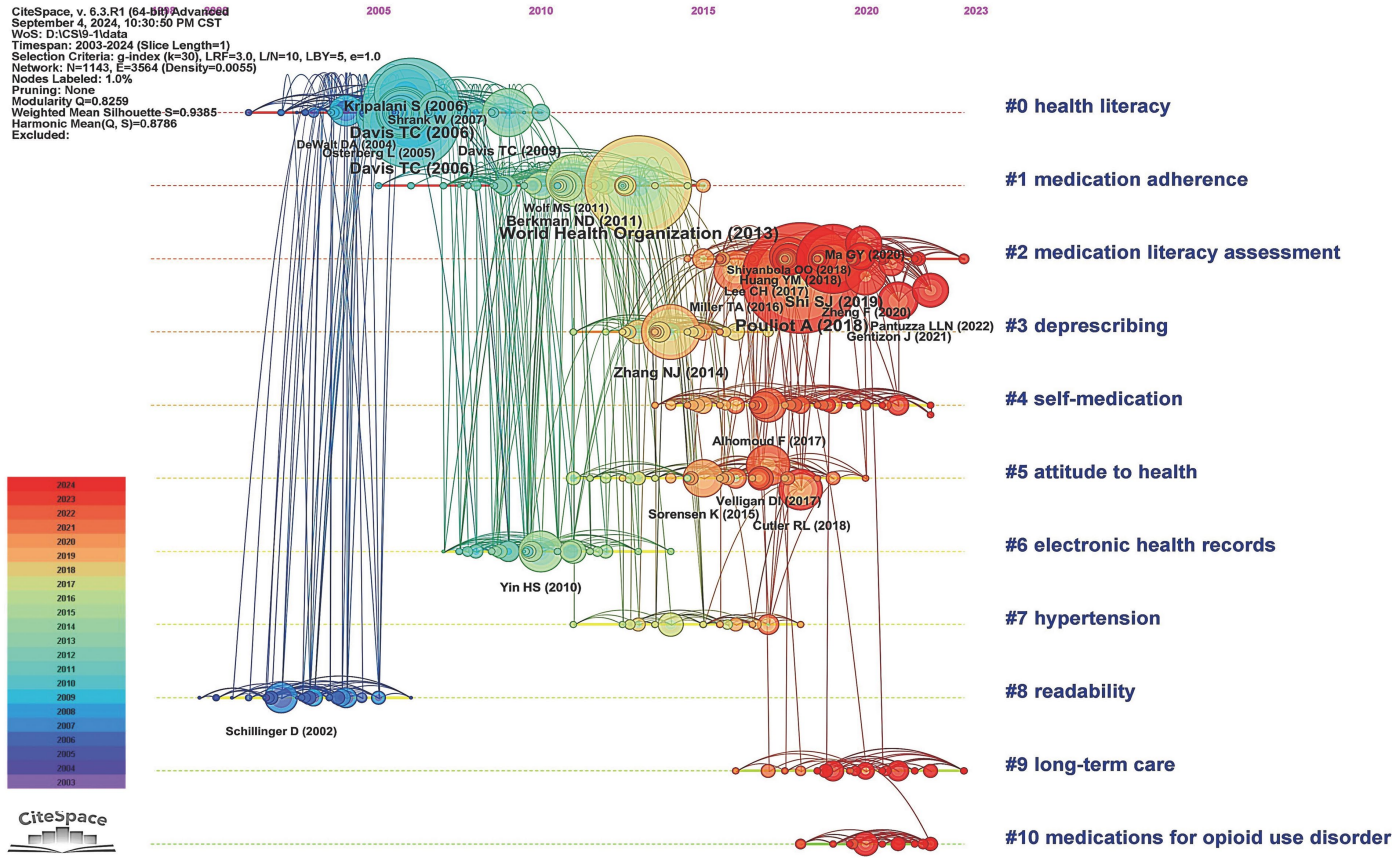
Timeline visualization of co-citation references network (2003–2024). A node represents a cited reference. The size of a node depends on its betweenness centrality. For each cluster, nodes are organized by their year of publication on horizontal lines. Nodes with large coloured tree rings are those with high betweenness centrality (external purple tree rings) and burst strength (central red tree rings). The color of lines indicate the time of links between nodes or between clusters.

The timeline map provided a visual representation of the duration and historical progression of each cluster, effectively capturing the trends mentioned earlier. It also allowed us to pinpoint the temporal placement of landmark publications. Notably, the most recent and dynamically active clusters in the analysis were cluster #2 (*“medication literacy assessment”*), cluster #4 (*“self-medication”*), cluster #5 (*“attitude to health”*), cluster #9 (*“long-term care”*), and cluster #10 (*“medications for opioid use disorder”*), indicating a growing research interest in these areas (Figure 3).

#### 3.2.2 Most cited references and transformative papers

Table 2 presented the top ten most cited references, which played a crucial role in shaping the intellectual foundations of the clustering studies. A comprehensive review of strategies to assess and improve medication adherence/compliance conducted by L. Osterberg et al. in 2005 emerged as the most co-cited paper, with 98 citations within our reference network (44). Notably, a cross-sectional investigation of drug literacy and comprehension of prescription drug labels authored by Davis TC et al. in the Annals of Internal Medicine received 91 co-citations within our network (37). It is noteworthy that these two publications demonstrated substantial citation bursts, with burst strengths of 6.01 and 13.20, respectively, indicating their potential to exert significant influence on ML research (Supplementary Table S1).

**Table 2:** The top 10 most cited references.

Co-citations	Author	Year	Title	Journal
98	Osterberg L	2005	Adherence to medication	New England Journal of Medicine
91	Davis Tc	2006	Literacy and misunderstanding prescription drug labels	Annals of Internal Medicine
84	Horne R	1999	The Beliefs about Medicines Questionnaire: The development and evaluation of a new method for assessing the cognitive representation of medication	Psychology & Health
70	Hogan Tp	1983	A self-report scale predictive of drug compliance in schizophrenics: reliability and discriminative validity	Psychological Medicine
70	Morisky De	1986	Concurrent and predictive validity of a self-reported measure of medication adherence	Medical Care
67	Davis Tc	2006	Low literacy impairs comprehension of prescription drug warning labels	Journal of General Internal Medicine
66	Berkman Nd	2011	Low health literacy and health outcomes: an updated systematic review	Annals of Internal Medicine
66	Davis Tc	1993	Rapid estimate of adult literacy in medicine: a shortened screening instrument	Family medicine
66	Morisky De	2008	Predictive validity of a medication adherence measure in an outpatient setting	The Journal of Clinical Hypertension
62	Horne R	1999	Patients’ beliefs about prescribed medicines and their role in adherence to treatment in chronic physical illness	Journal of Psychosomatic Research

Furthermore, a structural variation analysis was conducted to identify transformative papers that derived disciplinary evolution in the research field through interdisciplinary knowledge integration. Utilizing centrality divergence metrics, we identified three paradigm-shifting publications: an illustrated medication schedule developed by Kripalani S et al. for better understanding of prescription drugs (45), an investigation of the relationship between patient literacy level and self-reported HIV medication adherence (46), and a multicenter study conducted by Persell SD et al. of health literacy on medication reconciliation in ambulatory care (47). These papers have made significant contributions to the field and have been instrumental in advancing our understanding of ML research.

### 3.3 Analysis of co-occurring author keywords

**Figure 4:**
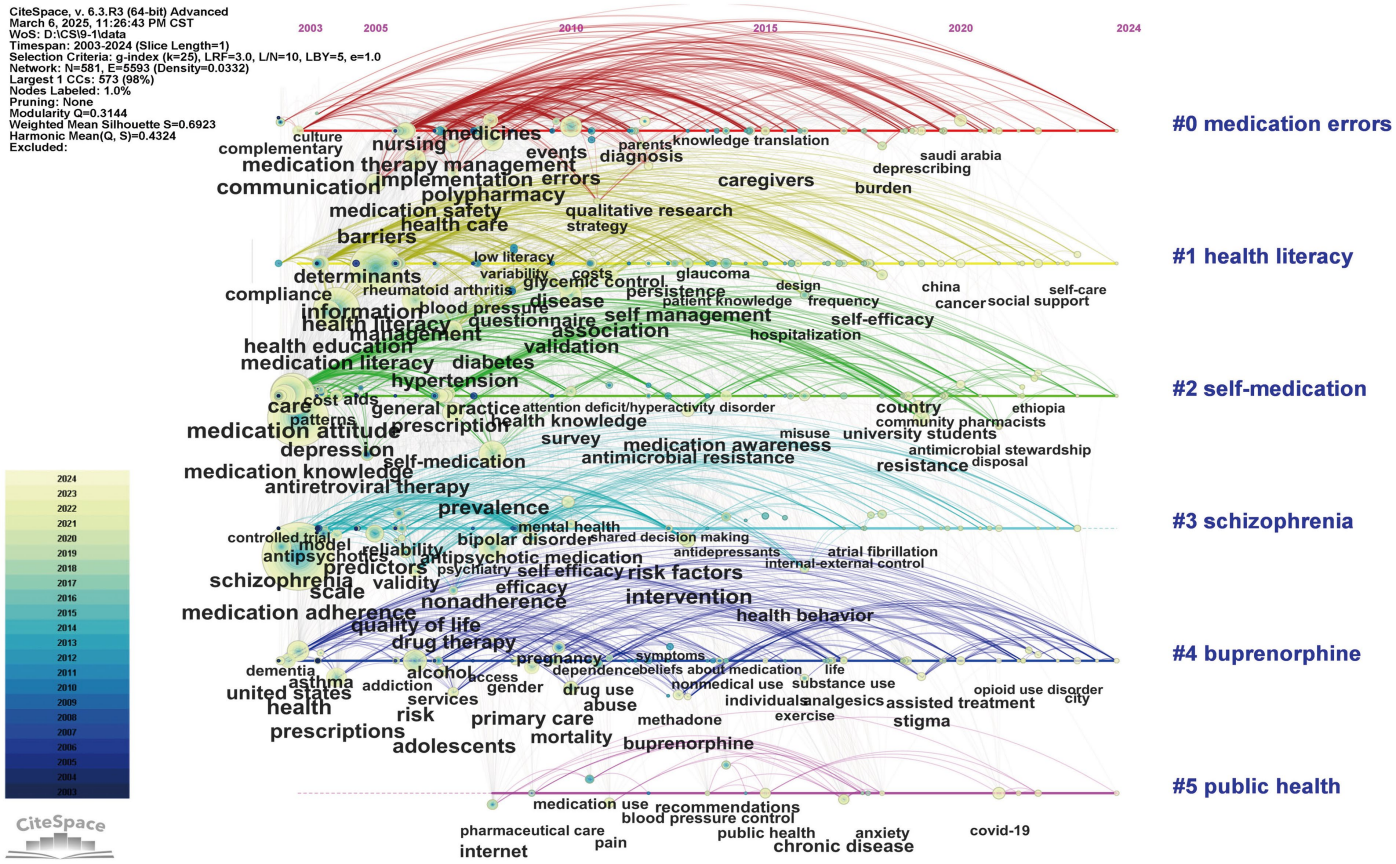
Timeline visualization of co-occurring keywords network (2003–2024). A node represents a keyword. The position of the node corresponds to the year of keyword occurrence. The size of a node is proportional to the frequency of its occurrence. The clusters are labelled in blue on the far right of the timeline map.

Figure 4 depicted a timeline visualization derived from the co-occurrence analysis of author keywords using CiteSpace, illustrating the evolution of thematic clusters in ML research. The keyword clustering exhibited robust validity, supported by high modularity and silhouette scores (Q = 0.3144; S = 0.6923), indicating well-defined and internally coherent groupings. The cluster labels were synthetically generated based on homogeneous, high-frequency keywords extracted from the citing literature. Six major clusters were identified (ranked by size): cluster #0 (*“medication errors”*; 134; S = 0.652; 2012), #1 (*“health literacy”*; 123; S = 0.671; 2011), #2 (*“self-medication”*; 107; S = 0.726; 2011), #3 (*“schizophrenia”*; 100; S = 0.742; 2009), #4 (*“buprenorphine”*; 90; S = 0.646; 2013), #5 (*“public health”*; 19; S = 0.884; 2016). We found that cluster #0, #1, #2, #3, and #4 showed extensive temporal spans (>20 years), suggesting their foundational role as core research themes within this domain. The concentration of high-frequency keywords in cluster #0, #1, and #3 during early stages indicated theoretical maturation in medication literacy, errors, and adherence research, with a visible translational shift toward clinical implementation studies in recent years. In contrast, the emergence of high-frequency keywords in cluster #2 and #4 reflected their status as burgeoning research frontiers, as evidenced by intensified scholarly activity recently (Figure 4).

**Figure 5:**
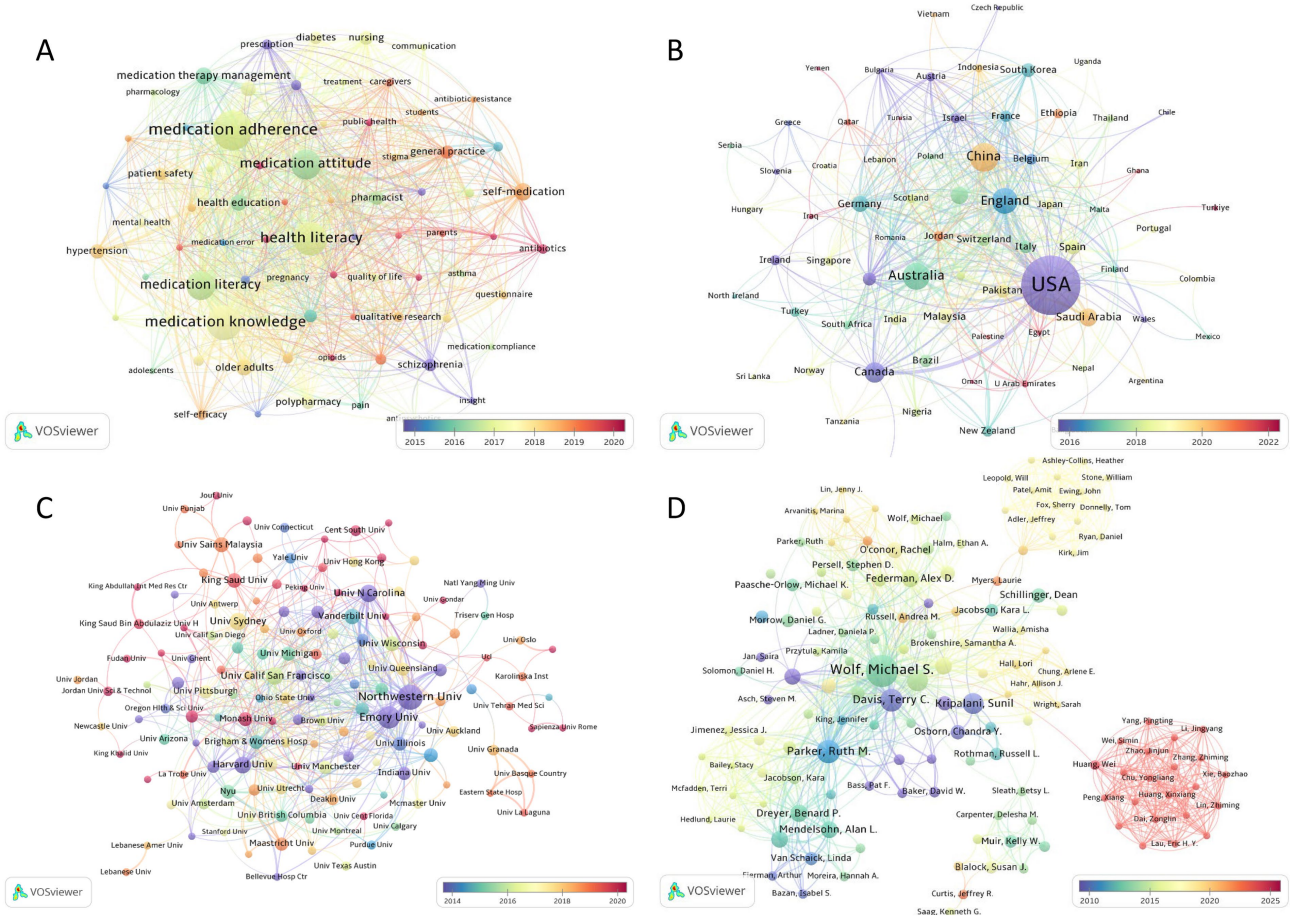
**(A)** Network of co-occurring author keywords; **(B)** Network of cooperation between countries; **(C)** Network of cooperation between institutions; **(D)** Network of cooperation between authors. The size of a node is proportional to the frequency of its occurrence. The color of the node corresponds to the average year of publication.

Furthermore, keywords were analyzed for burstiness to identify keywords that exhibited significant temporal fluctuations in academic attention (Supplementary Table S2). The keywords with the highest burst intensity were *quality* (strongest), *adherence*, and *nonadherence*. The most persistent keywords based on when the citation outbreak began were *comprehension*, *adverse drug events*, and *physicians*. Notably, *self-efficacy* and *resistance* emerged as areas of recent academic focus, showing continued prominence between 2018 and 2024. Additionally, VOSviewer software was to generate temporal overlay visualizations mapped to average publication year. The most frequently cited keywords encapsulated the major research trends-medication adherence, medication knowledge, health literacy, and medication attitudes-which were highly conceptually aligned with the thematic focus of this study (Figure 5A).

### 3.4 Analysis of collaboration networks across countries and institutions

Figure 5B displays the cooperation networks of countries, while Figure 5C shows the cooperation networks of institutions. In total, 72 countries or territories were captured in the analysis. The *United States of America* (USA) held a central position, with the highest number of publications (*n* = 768), followed by *China* with 181 publications and *Australia* with 164 publications. In terms of citations, the *USA* was also the most cited country (*n* = 20,363), followed by *England* (*n* = 3,281) and *Canada* (*n* = 2,325). Furthermore, VOSviewer identified 150 institutions from the dataset. *Northwestern University* emerged as both the most published institution (*n* = 62) and the most cited institution (*n* = 3,564). *Emory University* also produced 49 publications, while *Sydney University* had 32 publications. In terms of citations, *Emory University* ranked the second (*n* = 3,254), followed by *Louisiana State University* (*n* = 1729).

### 3.5 Analysis of co-authorship networks

A network of co-cited authors was established, demonstrating significant modularity and silhouette scores (Q = 6,128; S = 0.8674) (Supplementary Figure S2). Cluster #0, titled *“health literacy”*, emerged as the predominant thematic cluster, central to the network and encompassing research domains including medication adherence, attitudes, knowledge, errors, and self-management. The top three most cited authors were *Davis TC* (*n* = 192), *World Health Organization* (*n* = 188), and *Wolf MS* (*n* = 169). Analysis of betweenness centrality, metric reflecting authors’ roles in bridging network subfields, entified *Hogan TP* (centrality = 0.14), *Bandura A* (0.12), and *Cramer JA* (0.10) as key interdisciplinary connectors. *Kalichman SC* was identified as the highest citation burst intensity, indicating a significant increase in scholarly influence, and the most active contributor during the 2005–2011 period (Supplementary Table S3).

Furthermore, analysis of collaborative author networks revealed prominent collaborative clusters anchored by *Wolf MS*, *Horne R*, *Davis TC*, *Parker RM*, and *Kripalani S* (Figure 5D). These scholars occupied central network positions, driving both collaborative synergies and advancements in the field.

### 3.6 Analysis of journal occurrence and citations

**Figure 6:**
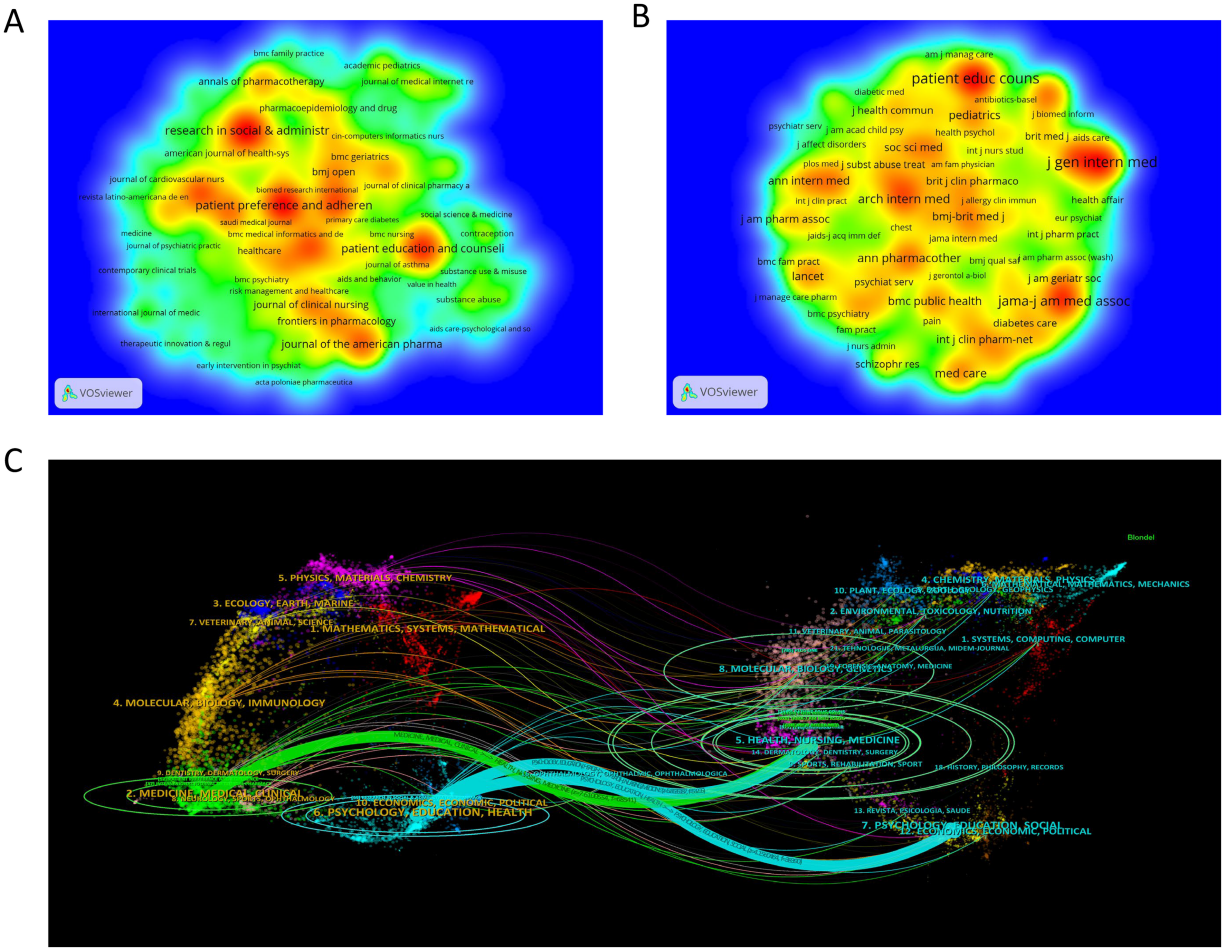
**(A)** Density map of journal publications; **(B)** Density map of cited journals; **(C)** Map of citation trajectories of citing and cited journals.

This study employed VOSviewer to construct journal co-occurrence (Figure 6A) and journal citation density maps (Figure 6B), systematically indicating the distribution characteristics of journals in ML research. *Research in Social & Administrative Pharmacy* (IF = 3.7, Q1) ranked first with 55 published papers, followed by *Patient Preference and Adherence* (*n* = 52, IF = 2.0, Q1) and *Patient Education and Counseling* (*n* = 46, IF = 2.9, Q1), collectively forming the core knowledge dissemination platforms in this domain. From an academic influence perspective, *Patient Education and Counseling* dominated with 1,180 total citations, followed by the *Journal of General Internal Medicine* (*n* = 1,151, IF = 4.3, Q1) and *JAMA-Journal of the American Medical Association* (*n* = 928, IF = 63.5, Q1), highlighting their disciplinary leadership. Dual-map overlay analysis further revealed knowledge flow patterns (Figure 6C). The citing journal cluster (left) and cited journal cluster (right) demonstrated two prominent knowledge transfer pathways: (1) Publications from *“Health/Nursing/Medicine”* journals primarily informed advancements in clinical medicine and health education research; (2) Outputs from *“Psychology/Education/Social”* journals were predominantly inherited by *“Psychology/Education/Health”* journals. This interdisciplinary citation paradigm underscores the dual attributes of ML research as clinical treatment and socio-educational relevance, providing theoretical guidance for journal selection strategies.

## 4 Discussion

### 4.1 Summary of the main findings

This study offered a comprehensive scientometric assessment of the global ML research landscape, delineating its intellectual structure, thematic evolution, and collaborative patterns over a 20-year period. While annual publication output remained modest, the field exhibited a marked growth trajectory, reflecting its rising scholarly focus. Analyses of co-cited literature networks and author keyword clusters revealed robust linkages across 11 and 10 thematic clusters, respectively, converged into three research themes: the relationship between ML and medication adherence, development of ML-specific assessment instruments, and investigations of psychosocial factors associated with ML. *The United States* served as the leading contributor nationally, with *Northwestern University* emerging as the most productive institutional entity. *Davis TC*, *Wolf MS*, and *Horne R* were identified as the most frequently cited authors, while *Wolf MS* demonstrated the highest publication productivity. The journals *Patient Education and Counseling*, *Journal of General Internal Medicine*, and *JAMA-Journal of the American Medical Association* ranked as the most influential outlets in the field.

### 4.2 Identification of research trends

The resulting co-citation reference network and author’s keyword analysis extracted three distinct major research trends in ML research from 2003 to 2024, which were also captured by the qualitative analysis of highly cited literature. The first research trend focused on the association between ML and medication adherence. The interplay between ML and medication adherence had emerged as a critical determinant of clinical outcomes. ML referred to a patient’s capacity to acquire, comprehend, and apply medication-related information, encompassing knowledge of drug nomenclature, dosing protocols, administration standards, and risk profiles (7). Medication knowledge represented the fundamental understanding of drug-related information (e.g., dosage, administration), whereas ML emphasized higher-order competencies in acquiring, evaluating, and applying such information in clinical practice (e.g., dose adjustment, adverse reaction identification) (48). Existed Studies had demonstrated that patients with adequate knowledge but insufficient literacy remained at significantly elevated risk of medication errors (48). Medication adherence was manifested as behavioral consistency with the prescribed treatment regimen (49). Previous evidence had demonstrated that ML positively modulated adherence through multilevel synergistic mechanisms (40). The first mechanism was cognitive reinforcement. Enhanced understanding of pharmacological mechanisms and long-term therapeutic necessity reduced self-discontinuation behaviors triggered by symptomatic relief. Patients might benefit from routine medication use reviews (MURs) with their healthcare providers to identify and address potential medication-related problems in advance (50). The second one was skill empowerment. Pharmaceutical care interventions, including regimen simplification (e.g., reduced dosing frequency) and visual medication aids (e.g., dosing calendars), mitigated non-adherence stemming from operational errors (51). Additionally, based on the Health Belief Model, ML increased people’s awareness of disease severity and therapeutic benefits, fostering a paradigm shift from passive compliance to active therapeutic engagement (52). This transformation proved particularly salient in chronic disease and mental health management (53). Attitudes and behaviors were identified as independent predictors of medication adherence (14, 54). While socioeconomic and polypharmacy challenges persisted as adherence inhibitors, integrative strategies combining ML interventions (e.g., personalized education, cost-reduction policy dissemination) and digital health technologies (e.g., medication reminder applications) demonstrated synergistic potential to counterbalance these impediments (55–58).

The second major and influential research trend involved the development of measurement tools for assessing ML. Scientifically validated and contextually appropriate assessment instruments constituted a critical prerequisite for both accurately evaluating individual ML levels and designing evidence-based intervention strategies. Given the multifactorial nature of ML, encompassing cognitive, behavioral, and sociocultural dimensions, its concept and evaluative criteria must be contextually adapted rather than universally standardized across various populations and healthcare contexts (7). As emphasized by Gentizon et al. (59), assessments required tailored calibration to align theoretical constructs of ML with practical measurement paradigms, ensuring congruence with specific demographic profiles and clinical settings. However, the heterogeneous quality of existing ML measurement instruments and the diverse emphases in their assessment scopes posed substantial challenges in the selection of ML assessment tools that were suitable for specific chronic disease patients (12, 19, 59). The unidimensional 14-item MedLitRxSE, as developed by Sauceda et al. (60), was presently the sole instrument formally recommended by the Agency for Healthcare Research and Quality (AHRQ) for evaluating ML among adult care recipients and their informal caregivers. This instrument exhibited reliable values, satisfactory content validity, structural validity, and internal consistency; however, its overall reliability remained uncertain. Additionally, the extensive content coverage of the C-MLSHP (14) and the Pharmacy Consumer Health Literacy Questionnaire (61), along with the satisfactory content validity of the PTHL-SR and MedLit-NSAID (19, 62), provided a robust foundation for the psychometric analysis of subsequent measurement instruments. The content validity of these instruments required further investigation, particularly with a focus on systematic engagement of target populations. Furthermore, previous studies had indicated that the PWMIL (63), the RALPH Interview Guide (64), and the Medication Literacy Questionnaire for Discharged Patients (65) provided uncertain evidence, necessitating further testing for both content and agency. Performance-based ML assessment instruments (e.g., MedLitRxSE) utilized standardized scenario testing to objectively quantify medication management competencies (e.g., dosage calculation, medication label interpretation), offering strong reliability and reproducibility (60). However, these tools might fail to fully capture patients’ adaptive capacities in real-world medication use contexts. Conversely, perception-based measures (e.g., the HeLMS questionnaire) assessed self-reported medication-taking confidence and challenges through subjective evaluations, which effectively identify experiential barriers but remain vulnerable to recall bias and social desirability effects (66). Adopting integrated methodologies in future research—combining performance-based tools to identify skill gaps with perception-based measures to uncover behavioral determinants—will be critical for comprehensive evaluation.

The development of ML were shaped by multidimensional psychosocial factors, centered on the dynamic interaction mechanisms between individuals and their environments (24). According to the Social Cognitive Theory (SCT) and Health Belief Model (HBM), patients’ medication literacy was not solely determined by cognitive ability, but also closely associated with psychological state, social support and cultural context (67–69). First, patients’ ability to interpret and integrate medication-related information was directly affected by education level (18). Due to barriers in understanding technical terminology or deficiencies in logical analysis, populations with a low education level often struggled to accurately assess medication risks and benefits. For example, previous studies revealed that, compared to 78% among highly educated groups, only 32% of chronic disease patients in low-income communities could correctly interpret dosage adjustment instructions on drug labels (70). Second, family and community support were important external resources for ML practice (71). Medication adherence can be increased by more than 40% through the proactive involvement of family members, including medication reminders, emotional reassurance, and behavioral monitoring (72). Conversely, social isolation or familial conflicts may contribute to medication discontinuation behaviors. For example, depressed patients who were lacking emotional support had a 2.3-fold higher risk of self-reducing antidepressant dosages (72). Third, patients’ perceptions of disease severity, treatment benefits, and self-management confidence constituted intrinsic drivers of ML (58, 59). Brod et al. found that psychological insulin resistance was associated with patients’ beliefs regarding diabetes and insulin, negative self-perceptions and attitudinal barriers, and fears of adverse outcomes and complications of insulin use, thereby contributing to patients’ reluctance to initiate and intensify treatment (73). In addition, the risk of medication use was exacerbated by financial stress and sensitivity to the cost of medication, which prompted some patients to purchase medication through informal sources or to self-adjust their dosage. Approximately half of patients with NCDs in developing countries were forced to reduce their use of prescription medications due to financial constraints (74).

Overall, the identified research trends were interconnected, forming a cyclical “assessment-mechanism-intervention” framework that collectively advanced ML research. The development of standardized ML assessment tools established a methodological foundation, enabling robust validation of the relationship between ML and medication adherence. Identification of the psychosocial factors served dual purposes: explaining individual variations in ML levels and enhancing the cultural relevance of assessment tools—both critical for designing personalized interventions. Importantly, the optimized interventions not only improved adherence but may also have their effectiveness moderated by psychosocial factors. The systematic associations represented the significant scientific value of ML research proceeding from assessment to intervention.

### 4.3 Outputs and influence networks

The analysis of research outputs and influence networks constitutes a secondary objective of this investigation, aiming to capture geographic distributions, identify gaps, and recognize high-impact countries, research groups, and authors within specific subjects. The presented collaborative networks, co-citation visualizations, and associated bibliometric indices, provide readers, particularly active researchers, with critical insights into the field’s epistemic architecture. At the national/institutional level, the *USA* and *Northwestern University* emerged as the foremost contributors in both publication volume and citations, which can be attributed to their top researchers and well-established biomedical foundations. Contrasted with the comparative lag of developing regions, the extensive collaboration in ML research in the Western countries and institutions reveals structural inequalities in medication-related health research globally. Substantive support for under-resourced nations and institutions is imperative to advance understanding of how various healthcare systems and sociocultural paradigms influence ML research. Furthermore, our co-cited author network highlights the significant contributions of *Davis TC* to the field, particularly in the relationship between ML and understanding of prescription drug labels (37, 38). While co-authorship networks provide limited proxy measures of scholarly influence, systematic examination of high-impact citations and transformative literature enables identification of field-shaping contributors. It warrants emphasis that journal rankings derived from WOSCC publication/citation counts constitute imperfect quality proxies. However, the analysis of co-cited journals does identify the most cited journals in a given research area, such as *Patient Education and Counseling* in our network, which are considered appropriate for specific topics. In addition, an emerging trend of cross-disciplinary research was observed in the ML field. Different domains, including clinical practice, social behavior, social education, and digital medications, were establishing a multidimensional ML-enhancement system. A paradigm shift of research mindset was required for investigators within this field.

### 4.4 Potential trends for future research

Acting as a pivotal bridge between patient cognition and medication behavior, ML is transitioning from a unidimensional analysis to a multidisciplinary exploration of dynamic mechanisms (75, 76). Future research should prioritize examining the impact of cultural heterogeneity on ML and the potential of digital interventions, such as AI-assisted medication guidance, to enhance adherence (77, 78). Cross-disciplinary collaborations, such as integrating psychology with pharmaceutical care, could optimize adherence strategies, particularly for vulnerable populations like older adults and individuals with limited education (79). Current assessment tools, such as MedLitRxSE, demonstrate limitations in dimensional coverage and technical adaptability within digital health contexts (59). To address these limitations, future efforts should focus on developing comprehensive scales that integrate functional, critical, interactive, and digital literacy dimensions, augmented by natural language processing technologies to enable real-time dynamic evaluations (77). For example, intelligent platforms utilizing computerized adaptive testing could incorporate electronic health record data to generate personalized feedback, thereby enhancing clinical utility (77). Additionally, the mediating and moderating roles of psychosocial factors, such as self-efficacy and social support, in the “ML to adherence” pathway require systematic validation (80). Mixed-methods approaches, combining longitudinal data with qualitative interviews, could elucidate the dynamic mediating effects of self-efficacy on ML-adherence relationships and the buffering role of familial support in low-education populations (24). Future studies must quantify the relative weights of psychosocial determinants and design precision interventions targeting at-risk subgroups, such as individuals with low literacy and ethnic minorities, to establish an ecological support network spanning individual, community, and policy levels. This integrated approach will advance the construction of a robust framework for enhancing ML, ultimately promoting equitable medication management and improved health outcomes.

### 4.5 Strengths and limitations

Compared to a narrative review, scientometric analysis provides a more systematic and comprehensive approach to mapping research landscapes, offering clinicians and researchers critical insights into emerging trends and intellectual structures. This method contributes to identifying underexplored scientific questions, thereby guiding the direction of future research efforts (25). Furthermore, it enables the identification of influential authors, journals, and institutions within the field of ML, fostering opportunities for collaboration and knowledge exchange across specialized research domains. However, several limitations of this study must be acknowledged. First, while co-citation analysis is an essential component of scientometric methods, it is susceptible to citation biases, including publication bias, self-citation, authorship bias, literature type bias, and journal impact factor bias, which may undermine the objectivity of the findings (32). Second, data collection was restricted to the SCIE and SSCI within the WOSCC, limiting the scope of retrieved publications. Other prominent databases, such as PubMed and Embase, which provide full-text references and citation lists, were excluded (31). Third, the co-citation network analysis focused solely on first authors, potentially overlooking the contributions of co-authors. Additionally, the keyword co-occurrence networks were susceptible to variations in keyword expressions, thus affecting cluster interpretation. Finally, the co-citation network’s ability to capture recent trends was constrained by the limited citation of newly published literature.

## 5 Conclusion

This first scientometric study provides a comprehensive analysis of the historical trends and research landscape of ML research, revealing sustained growth in scholarly output over two decades, with publication volumes peaking in 2022. The analysis identifies leading contributors, including the most productive countries, institutions, authors, and journals, while mapping thematic priorities such as the relationship between ML and medication adherence, development of ML-specific assessment instruments, and investigations of psychosocial factors associated with ML. The findings underscore the necessity for strengthened cross-institutional collaboration, particularly among European, U.S., and Chinese entities—to leverage the influence of key opinion leaders. By synthesizing current research trends and emerging frontiers, this work provides clinicians and researchers with an empirical foundation to guide future inquiries, while offering funding bodies strategic insights into priority areas.

## Data Availability

The raw data supporting the conclusions of this article will be made available by the authors, without undue reservation.
