# Supplementary material for: Knowledge domain and emerging trends in medication literacy research from 2003 to 2024: a scientometric and bibliometric analysis using CiteSpace and VOSviewer
Source: Front Public Health. 2025 Jun 24;13:1598482. doi: 10.3389/fpubh.2025.1598482 (PMC12234531; doi:10.3389/fpubh.2025.1598482)
Supplement: Supplementary file 1 [file Data_Sheet_1.docx]

**SUPPLEMENTARY MATERIALS**

**
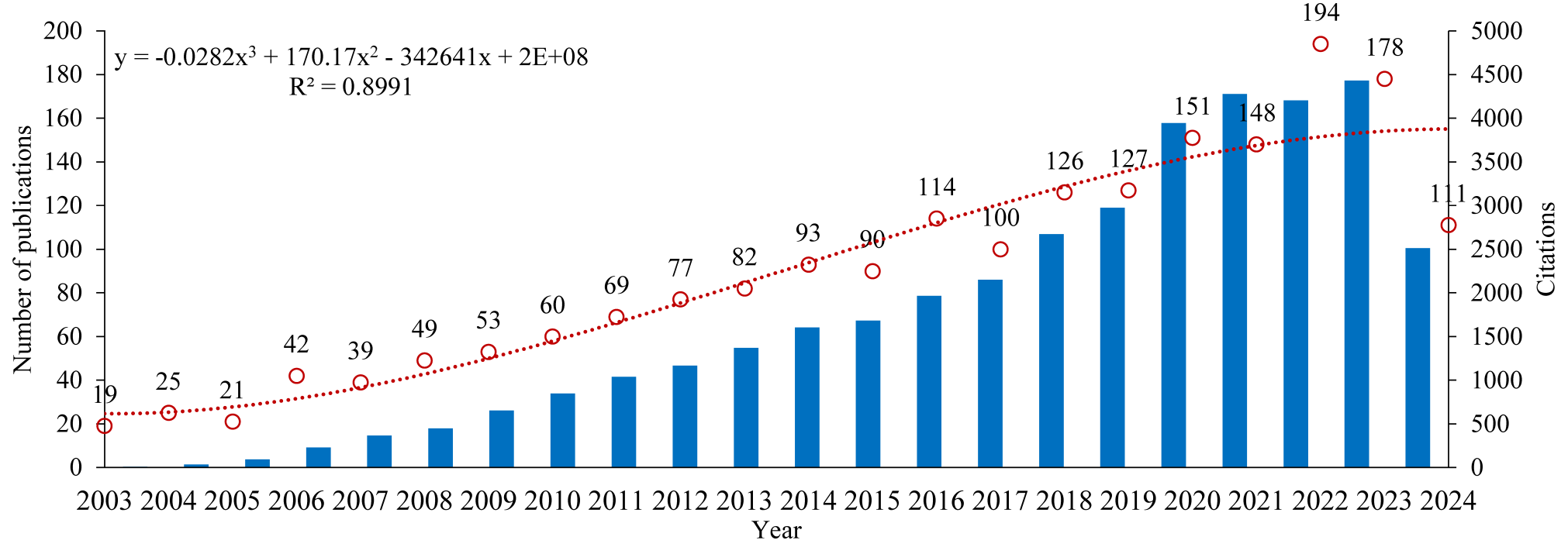
**

**Figure S1. Number of publications and citations per year**

**
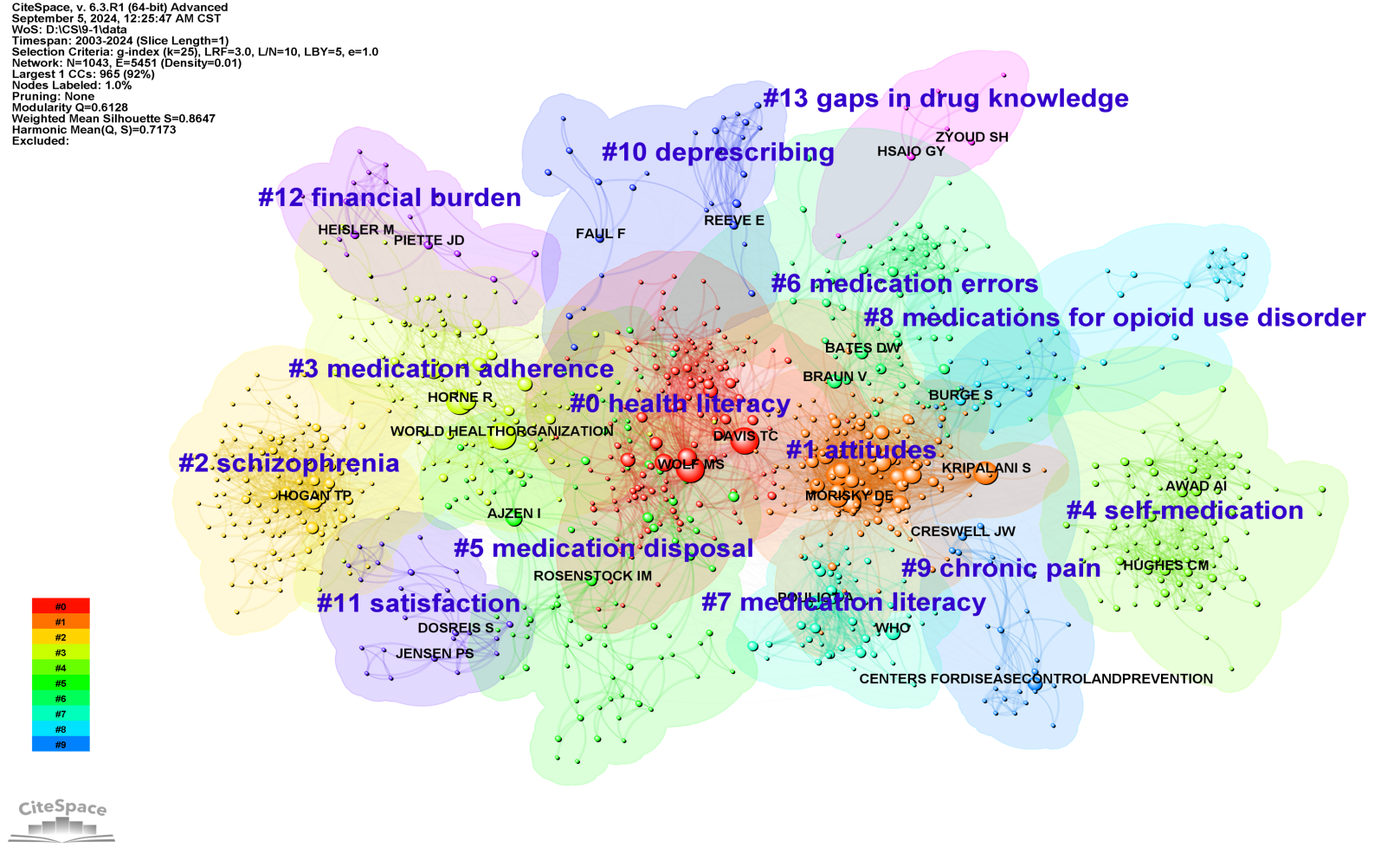
**

**Figure S2. Co-cited authors network and corresponding clustering visualization generated by CiteSpace.** Note: A node represents a cited author. The network is organized by the number of citations per node. The size of a node is proportional to its citations. Lines indicate co-citation relationships between authors. A large node indicates significant contributions and influence in the network.

**Table S1. Top 25 references with the strongest citation bursts (Minimum 4-year burst duration)**

| **References** | **Year** | **Strength** | **Begin** | **End** | **2003 - 2024** |
| --- | --- | --- | --- | --- | --- |
| Pouliot A, 2018, RES SOC ADMIN PHARM, V14, P797, DOI 10.1016/j.sapharm.2017.11.005 | 2018 | **13.28** | 2020 | 2024 | ▂▂▂▂▂▂▂▂▂▂▂▂▂▂▂▂▂▃▃▃▃▃ |
| World Health Organization, 2013, ADHERENCE TO LONG-TERM THERAPIES: EVIDENCE FOR ACTION, V0, P0 | 2013 | **13.28** | 2013 | 2018 | ▂▂▂▂▂▂▂▂▂▂▃▃▃▃▃▃▂▂▂▂▂▂ |
| Davis TC, 2006, J GEN INTERN MED, V21, P847, DOI 10.1111/j.1525-1497.2006.00529.x | 2006 | **13.2** | 2007 | 2011 | ▂▂▂▂▃▃▃▃▃▂▂▂▂▂▂▂▂▂▂▂▂▂ |
| Davis TC, 2006, ANN INTERN MED, V145, P887, DOI 10.7326/0003-4819-145-12-200612190-00144 | 2006 | **13.2** | 2007 | 2011 | ▂▂▂▂▃▃▃▃▃▂▂▂▂▂▂▂▂▂▂▂▂▂ |
| Kripalani S, 2006, J GEN INTERN MED, V21, P852, DOI 10.1111/j.1525-1497.2006.00536.x | 2006 | **8.59** | 2007 | 2011 | ▂▂▂▂▃▃▃▃▃▂▂▂▂▂▂▂▂▂▂▂▂▂ |
| Wolf MS, 2007, PATIENT EDUC COUNS, V67, P293, DOI 10.1016/j.pec.2007.03.024 | 2007 | **8.16** | 2009 | 2012 | ▂▂▂▂▂▂▃▃▃▃▂▂▂▂▂▂▂▂▂▂▂▂ |
| Gazmararian JA, 2006, J GEN INTERN MED, V21, P1215, DOI 10.1111/j.1525-1497.2006.00591.x | 2006 | **7.57** | 2007 | 2011 | ▂▂▂▂▃▃▃▃▃▂▂▂▂▂▂▂▂▂▂▂▂▂ |
| Berkman ND, 2011, ANN INTERN MED, V155, P97, DOI 10.7326/0003-4819-155-2-201107190-00005 | 2011 | **7.07** | 2012 | 2016 | ▂▂▂▂▂▂▂▂▂▃▃▃▃▃▂▂▂▂▂▂▂▂ |
| Wolf MS, 2006, AM J HEALTH-SYST PH, V63, P1048, DOI 10.2146/ajhp050469 | 2006 | **7.06** | 2007 | 2011 | ▂▂▂▂▃▃▃▃▃▂▂▂▂▂▂▂▂▂▂▂▂▂ |
| Davis TC, 2009, J GEN INTERN MED, V24, P57, DOI 10.1007/s11606-008-0833-4 | 2009 | **6.7** | 2010 | 2014 | ▂▂▂▂▂▂▂▃▃▃▃▃▂▂▂▂▂▂▂▂▂▂ |
| Wolf MS, 2006, PATIENT EDUC COUNS, V62, P316, DOI 10.1016/j.pec.2006.06.010 | 2006 | **6.55** | 2007 | 2011 | ▂▂▂▂▃▃▃▃▃▂▂▂▂▂▂▂▂▂▂▂▂▂ |
| Yin HS, 2010, ARCH PEDIAT ADOL MED, V164, P181, DOI 10.1001/archpediatrics.2009.269 | 2010 | **6.2** | 2012 | 2015 | ▂▂▂▂▂▂▂▂▂▃▃▃▃▂▂▂▂▂▂▂▂▂ |
| Shrank WH, 2007, ARCH INTERN MED, V167, P1760, DOI 10.1001/archinte.167.16.1760 | 2007 | **6.1** | 2009 | 2012 | ▂▂▂▂▂▂▃▃▃▃▂▂▂▂▂▂▂▂▂▂▂▂ |
| Osterberg L, 2005, NEW ENGL J MED, V353, P487, DOI 10.1056/NEJMra050100 | 2005 | **6.01** | 2006 | 2010 | ▂▂▂▃▃▃▃▃▂▂▂▂▂▂▂▂▂▂▂▂▂▂ |
| Miller TA, 2016, PATIENT EDUC COUNS, V99, P1079, DOI 10.1016/j.pec.2016.01.020 | 2016 | **5.52** | 2018 | 2021 | ▂▂▂▂▂▂▂▂▂▂▂▂▂▂▂▃▃▃▃▂▂▂ |
| Tarn DM, 2006, ARCH INTERN MED, V166, P1855, DOI 10.1001/archinte.166.17.1855 | 2006 | **5.46** | 2007 | 2010 | ▂▂▂▂▃▃▃▃▂▂▂▂▂▂▂▂▂▂▂▂▂▂ |
| Shrank W, 2007, ANN PHARMACOTHER, V41, P783, DOI 10.1345/aph.1H582 | 2007 | **5.08** | 2009 | 2012 | ▂▂▂▂▂▂▃▃▃▃▂▂▂▂▂▂▂▂▂▂▂▂ |
| DeWalt DA, 2004, J GEN INTERN MED, V19, P1228, DOI 10.1111/j.1525-1497.2004.40153.x | 2004 | **4.89** | 2006 | 2009 | ▂▂▂▃▃▃▃▂▂▂▂▂▂▂▂▂▂▂▂▂▂▂ |
| Kripalani S, 2007, PATIENT EDUC COUNS, V66, P368, DOI 10.1016/j.pec.2007.01.020 | 2007 | **4.57** | 2009 | 2012 | ▂▂▂▂▂▂▃▃▃▃▂▂▂▂▂▂▂▂▂▂▂▂ |
| Huang YM, 2018, PATIENT EDUC COUNS, V101, P1906, DOI 10.1016/j.pec.2018.06.010 | 2018 | **4.51** | 2020 | 2024 | ▂▂▂▂▂▂▂▂▂▂▂▂▂▂▂▂▂▃▃▃▃▃ |
| Alhomoud F, 2017, INT J INFECT DIS, V57, P3, DOI 10.1016/j.ijid.2017.01.014 | 2017 | **4.41** | 2019 | 2022 | ▂▂▂▂▂▂▂▂▂▂▂▂▂▂▂▂▃▃▃▃▂▂ |
| Cutler RL, 2018, BMJ OPEN, V8, P0, DOI 10.1136/bmjopen-2017-016982 | 2018 | **4.3** | 2019 | 2024 | ▂▂▂▂▂▂▂▂▂▂▂▂▂▂▂▂▃▃▃▃▃▃ |
| Wolf MS, 2009, MED CARE, V47, P370, DOI 10.1097/MLR.0b013e31818af91a | 2009 | **4.3** | 2010 | 2014 | ▂▂▂▂▂▂▂▃▃▃▃▃▂▂▂▂▂▂▂▂▂▂ |
| Wolf MS, 2011, MED CARE, V49, P96, DOI 10.1097/MLR.0b013e3181f38174 | 2011 | **4.23** | 2012 | 2016 | ▂▂▂▂▂▂▂▂▂▃▃▃▃▃▂▂▂▂▂▂▂▂ |
| Kripalani S, 2010, PATIENT EDUC COUNS, V81, P177, DOI 10.1016/j.pec.2010.04.030 | 2010 | **4.13** | 2012 | 2015 | ▂▂▂▂▂▂▂▂▂▃▃▃▃▂▂▂▂▂▂▂▂▂ |

**Table S2. Burstness analysis of author keywords**

| **A. Top 20 keywords with the strongest strength of citation bursts**  **(Minimum 5-year burst duration)** | | | | | |
| --- | --- | --- | --- | --- | --- |
| **Keywords** | **Year** | **Strength** | **Begin** | **End** | **2003 - 2024** |
| quality | 2007 | **7.54** | 2011 | 2015 | ▂▂▂▂▂▂▂▂▃▃▃▃▃▂▂▂▂▂▂▂▂▂ |
| compliance | 2003 | **6.71** | 2003 | 2009 | ▃▃▃▃▃▃▃▂▂▂▂▂▂▂▂▂▂▂▂▂▂▂ |
| noncompliance | 2004 | **6.54** | 2004 | 2011 | ▂▃▃▃▃▃▃▃▃▂▂▂▂▂▂▂▂▂▂▂▂▂ |
| therapy | 2003 | **6.45** | 2010 | 2016 | ▂▂▂▂▂▂▂▃▃▃▃▃▃▃▂▂▂▂▂▂▂▂ |
| resistance | 2019 | **5.93** | 2019 | 2024 | ▂▂▂▂▂▂▂▂▂▂▂▂▂▂▂▂▃▃▃▃▃▃ |
| skills | 2007 | **5.79** | 2007 | 2013 | ▂▂▂▂▃▃▃▃▃▃▃▂▂▂▂▂▂▂▂▂▂▂ |
| comprehension | 2004 | **5.66** | 2004 | 2013 | ▂▃▃▃▃▃▃▃▃▃▃▂▂▂▂▂▂▂▂▂▂▂ |
| physicians | 2008 | **5.53** | 2008 | 2016 | ▂▂▂▂▂▃▃▃▃▃▃▃▃▃▂▂▂▂▂▂▂▂ |
| women | 2010 | **4.54** | 2013 | 2017 | ▂▂▂▂▂▂▂▂▂▂▃▃▃▃▃▂▂▂▂▂▂▂ |
| self management | 2013 | **4.1** | 2013 | 2017 | ▂▂▂▂▂▂▂▂▂▂▃▃▃▃▃▂▂▂▂▂▂▂ |
| chronic disease | 2017 | **3.81** | 2017 | 2022 | ▂▂▂▂▂▂▂▂▂▂▂▂▂▂▃▃▃▃▃▃▂▂ |
| self-efficacy | 2018 | **3.69** | 2018 | 2024 | ▂▂▂▂▂▂▂▂▂▂▂▂▂▂▂▃▃▃▃▃▃▃ |
| patient knowledge | 2014 | **3.67** | 2014 | 2018 | ▂▂▂▂▂▂▂▂▂▂▂▃▃▃▃▃▂▂▂▂▂▂ |
| burden | 2018 | **3.65** | 2018 | 2022 | ▂▂▂▂▂▂▂▂▂▂▂▂▂▂▂▃▃▃▃▃▂▂ |
| subjective response | 2005 | **3.6** | 2005 | 2009 | ▂▂▃▃▃▃▃▂▂▂▂▂▂▂▂▂▂▂▂▂▂▂ |
| acetaminophen | 2006 | **3.34** | 2012 | 2018 | ▂▂▂▂▂▂▂▂▂▃▃▃▃▃▃▃▂▂▂▂▂▂ |
| predictors | 2005 | **3.31** | 2005 | 2010 | ▂▂▃▃▃▃▃▃▂▂▂▂▂▂▂▂▂▂▂▂▂▂ |
| low literacy | 2009 | **3.28** | 2009 | 2014 | ▂▂▂▂▂▂▃▃▃▃▃▃▂▂▂▂▂▂▂▂▂▂ |
| outpatients | 2009 | **3.14** | 2009 | 2013 | ▂▂▂▂▂▂▃▃▃▃▃▂▂▂▂▂▂▂▂▂▂▂ |
| adverse drug events | 2006 | **3.08** | 2006 | 2014 | ▂▂▂▃▃▃▃▃▃▃▃▃▂▂▂▂▂▂▂▂▂▂ |

| **B. Top 20 keywords with the strongest beginning of citation busts**  **(Minimum 5-year burst duration)** | | | | | |
| --- | --- | --- | --- | --- | --- |
| **Keywords** | **Year** | **Strength** | **Begin** | **End** | **2003 - 2024** |
| compliance | 2003 | 6.71 | **2003** | 2009 | ▃▃▃▃▃▃▃▂▂▂▂▂▂▂▂▂▂▂▂▂▂▂ |
| noncompliance | 2004 | 6.54 | **2004** | 2011 | ▂▃▃▃▃▃▃▃▃▂▂▂▂▂▂▂▂▂▂▂▂▂ |
| comprehension | 2004 | 5.66 | **2004** | 2013 | ▂▃▃▃▃▃▃▃▃▃▃▂▂▂▂▂▂▂▂▂▂▂ |
| subjective response | 2005 | 3.6 | **2005** | 2009 | ▂▂▃▃▃▃▃▂▂▂▂▂▂▂▂▂▂▂▂▂▂▂ |
| predictors | 2005 | 3.31 | **2005** | 2010 | ▂▂▃▃▃▃▃▃▂▂▂▂▂▂▂▂▂▂▂▂▂▂ |
| adverse drug events | 2006 | 3.08 | **2006** | 2014 | ▂▂▂▃▃▃▃▃▃▃▃▃▂▂▂▂▂▂▂▂▂▂ |
| skills | 2007 | 5.79 | **2007** | 2013 | ▂▂▂▂▃▃▃▃▃▃▃▂▂▂▂▂▂▂▂▂▂▂ |
| physicians | 2008 | 5.53 | **2008** | 2016 | ▂▂▂▂▂▃▃▃▃▃▃▃▃▃▂▂▂▂▂▂▂▂ |
| low literacy | 2009 | 3.28 | **2009** | 2014 | ▂▂▂▂▂▂▃▃▃▃▃▃▂▂▂▂▂▂▂▂▂▂ |
| outpatients | 2009 | 3.14 | **2009** | 2013 | ▂▂▂▂▂▂▃▃▃▃▃▂▂▂▂▂▂▂▂▂▂▂ |
| therapy | 2003 | 6.45 | **2010** | 2016 | ▂▂▂▂▂▂▂▃▃▃▃▃▃▃▂▂▂▂▂▂▂▂ |
| quality | 2007 | 7.54 | **2011** | 2015 | ▂▂▂▂▂▂▂▂▃▃▃▃▃▂▂▂▂▂▂▂▂▂ |
| acetaminophen | 2006 | 3.34 | **2012** | 2018 | ▂▂▂▂▂▂▂▂▂▃▃▃▃▃▃▃▂▂▂▂▂▂ |
| women | 2010 | 4.54 | **2013** | 2017 | ▂▂▂▂▂▂▂▂▂▂▃▃▃▃▃▂▂▂▂▂▂▂ |
| self management | 2013 | 4.1 | **2013** | 2017 | ▂▂▂▂▂▂▂▂▂▂▃▃▃▃▃▂▂▂▂▂▂▂ |
| patient knowledge | 2014 | 3.67 | **2014** | 2018 | ▂▂▂▂▂▂▂▂▂▂▂▃▃▃▃▃▂▂▂▂▂▂ |
| chronic disease | 2017 | 3.81 | **2017** | 2022 | ▂▂▂▂▂▂▂▂▂▂▂▂▂▂▃▃▃▃▃▃▂▂ |
| self-efficacy | 2018 | 3.69 | **2018** | 2024 | ▂▂▂▂▂▂▂▂▂▂▂▂▂▂▂▃▃▃▃▃▃▃ |
| burden | 2018 | 3.65 | **2018** | 2022 | ▂▂▂▂▂▂▂▂▂▂▂▂▂▂▂▃▃▃▃▃▂▂ |
| resistance | 2019 | 5.93 | **2019** | 2024 | ▂▂▂▂▂▂▂▂▂▂▂▂▂▂▂▂▃▃▃▃▃▃ |

**Table S3. Burstness analysis of cited authors**

| 1. **Top 25 cited authors with the strongest strength of citation bursts**   **(Minimum 5year burst duration)** | | | | | |
| --- | --- | --- | --- | --- | --- |
| **Cited Authors** | **Year** | **Strength** | **Begin** | **End** | **2003 - 2024** |
| KALICHMAN SC | 2005 | **13.43** | 2005 | 2011 | ▂▂▃▃▃▃▃▃▃▂▂▂▂▂▂▂▂▂▂▂▂▂ |
| SORENSEN K | 2018 | **12.36** | 2019 | 2024 | ▂▂▂▂▂▂▂▂▂▂▂▂▂▂▂▂▃▃▃▃▃▃ |
| SCHILLINGER D | 2005 | **11.16** | 2005 | 2014 | ▂▂▃▃▃▃▃▃▃▃▃▃▂▂▂▂▂▂▂▂▂▂ |
| WILLIAMS MV | 2005 | **10.36** | 2005 | 2016 | ▂▂▃▃▃▃▃▃▃▃▃▃▃▃▂▂▂▂▂▂▂▂ |
| ZHANG NJ | 2017 | **8.8** | 2017 | 2021 | ▂▂▂▂▂▂▂▂▂▂▂▂▂▂▃▃▃▃▃▂▂▂ |
| KATZ MG | 2009 | **8.54** | 2009 | 2016 | ▂▂▂▂▂▂▃▃▃▃▃▃▃▃▂▂▂▂▂▂▂▂ |
| SHRANK WH | 2009 | **8.25** | 2009 | 2017 | ▂▂▂▂▂▂▃▃▃▃▃▃▃▃▃▂▂▂▂▂▂▂ |
| GAZMARARIAN JA | 2006 | **8.08** | 2006 | 2015 | ▂▂▂▃▃▃▃▃▃▃▃▃▃▂▂▂▂▂▂▂▂▂ |
| KEMP R | 2003 | **7.74** | 2003 | 2010 | ▃▃▃▃▃▃▃▃▂▂▂▂▂▂▂▂▂▂▂▂▂▂ |
| PARKER RM | 2005 | **7.71** | 2005 | 2016 | ▂▂▃▃▃▃▃▃▃▃▃▃▃▃▂▂▂▂▂▂▂▂ |
| HOUTS PS | 2005 | **7.51** | 2005 | 2013 | ▂▂▃▃▃▃▃▃▃▃▃▂▂▂▂▂▂▂▂▂▂▂ |
| WALLACE LS | 2011 | **7.45** | 2011 | 2015 | ▂▂▂▂▂▂▂▂▃▃▃▃▃▂▂▂▂▂▂▂▂▂ |
| DAVIS TC | 2005 | **7.39** | 2006 | 2013 | ▂▂▂▃▃▃▃▃▃▃▃▂▂▂▂▂▂▂▂▂▂▂ |
| OSBORN CY | 2013 | **6.87** | 2013 | 2020 | ▂▂▂▂▂▂▂▂▂▂▃▃▃▃▃▃▃▃▂▂▂▂ |
| BROWN MT | 2012 | **6.6** | 2017 | 2021 | ▂▂▂▂▂▂▂▂▂▂▂▂▂▂▃▃▃▃▃▂▂▂ |
| *I MED | 2006 | **6.57** | 2006 | 2012 | ▂▂▂▃▃▃▃▃▃▃▂▂▂▂▂▂▂▂▂▂▂▂ |
| OSTERBERG L | 2007 | **6.22** | 2011 | 2017 | ▂▂▂▂▂▂▂▂▃▃▃▃▃▃▃▂▂▂▂▂▂▂ |
| GANDHI TK | 2006 | **6.21** | 2006 | 2010 | ▂▂▂▃▃▃▃▃▂▂▂▂▂▂▂▂▂▂▂▂▂▂ |
| MORRIS LA | 2004 | **6.15** | 2004 | 2009 | ▂▃▃▃▃▃▃▂▂▂▂▂▂▂▂▂▂▂▂▂▂▂ |
| HO PM | 2011 | **6.1** | 2014 | 2019 | ▂▂▂▂▂▂▂▂▂▂▂▃▃▃▃▃▃▂▂▂▂▂ |
| WORLD HEALTH ORGANIZATION | 2012 | **5.93** | 2019 | 2024 | ▂▂▂▂▂▂▂▂▂▂▂▂▂▂▂▂▃▃▃▃▃▃ |
| DEWALT DA | 2006 | **5.93** | 2006 | 2014 | ▂▂▂▃▃▃▃▃▃▃▃▃▂▂▂▂▂▂▂▂▂▂ |
| BATES DW | 2006 | **5.88** | 2006 | 2014 | ▂▂▂▃▃▃▃▃▃▃▃▃▂▂▂▂▂▂▂▂▂▂ |
| FENTON WS | 2003 | **5.68** | 2003 | 2010 | ▃▃▃▃▃▃▃▃▂▂▂▂▂▂▂▂▂▂▂▂▂▂ |
| LEAPE LL | 2006 | **5.5** | 2006 | 2015 | ▂▂▂▃▃▃▃▃▃▃▃▃▃▂▂▂▂▂▂▂▂▂ |

| **B. Top 25 cited authors with the strongest beginning of citation bursts**  **(Minimum 5year burst duration)** | | | | | |
| --- | --- | --- | --- | --- | --- |
| **Cited Authors** | **Year** | **Strength** | **Begin** | **End** | **2003 - 2024** |
| KEMP R | 2003 | 7.74 | **2003** | 2010 | ▃▃▃▃▃▃▃▃▂▂▂▂▂▂▂▂▂▂▂▂▂▂ |
| FENTON WS | 2003 | 5.68 | **2003** | 2010 | ▃▃▃▃▃▃▃▃▂▂▂▂▂▂▂▂▂▂▂▂▂▂ |
| MORRIS LA | 2004 | 6.15 | **2004** | 2009 | ▂▃▃▃▃▃▃▂▂▂▂▂▂▂▂▂▂▂▂▂▂▂ |
| KALICHMAN SC | 2005 | 13.43 | **2005** | 2011 | ▂▂▃▃▃▃▃▃▃▂▂▂▂▂▂▂▂▂▂▂▂▂ |
| SCHILLINGER D | 2005 | 11.16 | **2005** | 2014 | ▂▂▃▃▃▃▃▃▃▃▃▃▂▂▂▂▂▂▂▂▂▂ |
| WILLIAMS MV | 2005 | 10.36 | **2005** | 2016 | ▂▂▃▃▃▃▃▃▃▃▃▃▃▃▂▂▂▂▂▂▂▂ |
| PARKER RM | 2005 | 7.71 | **2005** | 2016 | ▂▂▃▃▃▃▃▃▃▃▃▃▃▃▂▂▂▂▂▂▂▂ |
| HOUTS PS | 2005 | 7.51 | **2005** | 2013 | ▂▂▃▃▃▃▃▃▃▃▃▂▂▂▂▂▂▂▂▂▂▂ |
| GAZMARARIAN JA | 2006 | 8.08 | **2006** | 2015 | ▂▂▂▃▃▃▃▃▃▃▃▃▃▂▂▂▂▂▂▂▂▂ |
| DAVIS TC | 2005 | 7.39 | **2006** | 2013 | ▂▂▂▃▃▃▃▃▃▃▃▂▂▂▂▂▂▂▂▂▂▂ |
| *I MED | 2006 | 6.57 | **2006** | 2012 | ▂▂▂▃▃▃▃▃▃▃▂▂▂▂▂▂▂▂▂▂▂▂ |
| GANDHI TK | 2006 | 6.21 | **2006** | 2010 | ▂▂▂▃▃▃▃▃▂▂▂▂▂▂▂▂▂▂▂▂▂▂ |
| DEWALT DA | 2006 | 5.93 | **2006** | 2014 | ▂▂▂▃▃▃▃▃▃▃▃▃▂▂▂▂▂▂▂▂▂▂ |
| BATES DW | 2006 | 5.88 | **2006** | 2014 | ▂▂▂▃▃▃▃▃▃▃▃▃▂▂▂▂▂▂▂▂▂▂ |
| LEAPE LL | 2006 | 5.5 | **2006** | 2015 | ▂▂▂▃▃▃▃▃▃▃▃▃▃▂▂▂▂▂▂▂▂▂ |
| KATZ MG | 2009 | 8.54 | **2009** | 2016 | ▂▂▂▂▂▂▃▃▃▃▃▃▃▃▂▂▂▂▂▂▂▂ |
| SHRANK WH | 2009 | 8.25 | **2009** | 2017 | ▂▂▂▂▂▂▃▃▃▃▃▃▃▃▃▂▂▂▂▂▂▂ |
| WALLACE LS | 2011 | 7.45 | **2011** | 2015 | ▂▂▂▂▂▂▂▂▃▃▃▃▃▂▂▂▂▂▂▂▂▂ |
| OSTERBERG L | 2007 | 6.22 | **2011** | 2017 | ▂▂▂▂▂▂▂▂▃▃▃▃▃▃▃▂▂▂▂▂▂▂ |
| OSBORN CY | 2013 | 6.87 | **2013** | 2020 | ▂▂▂▂▂▂▂▂▂▂▃▃▃▃▃▃▃▃▂▂▂▂ |
| HO PM | 2011 | 6.1 | **2014** | 2019 | ▂▂▂▂▂▂▂▂▂▂▂▃▃▃▃▃▃▂▂▂▂▂ |
| ZHANG NJ | 2017 | 8.8 | **2017** | 2021 | ▂▂▂▂▂▂▂▂▂▂▂▂▂▂▃▃▃▃▃▂▂▂ |
| BROWN MT | 2012 | 6.6 | **2017** | 2021 | ▂▂▂▂▂▂▂▂▂▂▂▂▂▂▃▃▃▃▃▂▂▂ |
| SORENSEN K | 2018 | 12.36 | **2019** | 2024 | ▂▂▂▂▂▂▂▂▂▂▂▂▂▂▂▂▃▃▃▃▃▃ |
| WORLD HEALTH ORGANIZATION | 2012 | 5.93 | **2019** | 2024 | ▂▂▂▂▂▂▂▂▂▂▂▂▂▂▂▂▃▃▃▃▃▃ |
